# Differences in response to dienogest therapy among different phenotypes of endometriosis: A single-center retrospective cohort analysis

**DOI:** 10.1097/MD.0000000000047624

**Published:** 2026-02-13

**Authors:** Jixiao Liu, Dandan Wu, Jianli Sun

**Affiliations:** a Department of Gynecology, Tianjin Central Hospital of Gynecology Obstetrics, Tianjin City, China.

**Keywords:** dienogest, endometriosis, pain, phenotype, retrospective studies, treatment outcome

## Abstract

This study aims to investigate the differences in response to dienogest (DNG) therapy among patients with 3 different phenotypes of endometriosis: ovarian endometrioma (OMA), superficial peritoneal endometriosis (SUP), and deep infiltrating endometriosis (DIE). This study was a single-center retrospective cohort analysis. A total of 501 consecutive patients with endometriosis diagnosed and treated in our hospital from January 2023 to January 2024 were enrolled and divided into the OMA group (n = 276), SUP group (n = 125), and DIE group (n = 100) based on imaging/surgical phenotypes. All patients received monotherapy with DNG (2 mg/day) for at least 6 months. Differences in pain visual analog scale (VAS) scores, lesion size, serum CA125 levels, quality of life (36-Item Short Form Health Survey), and adverse reactions before and after treatment were compared among the 3 groups. Multivariate logistic regression analysis was used to identify independent influencing factors for significant pain relief (defined as ≥50% reduction in VAS score). At baseline, disease duration, revised American Society for Reproductive Medicine stage and score, CA125 levels, and pain intensity in the DIE group were significantly higher than those in the OMA and SUP groups (*P* < .001). After 6 months of treatment, pain VAS scores, lesion size, and CA125 levels significantly decreased from baseline in all 3 groups (*P* < .001). Intergroup comparisons showed statistically significant differences in the reduction of VAS scores (ΔVAS) for dysmenorrhea and chronic pelvic pain (*P* < .01), with the SUP group showing the smallest improvement. Meanwhile, the lesion reduction rate in the OMA group was significantly higher than that in the SUP and DIE groups (47.5% vs 39.4% vs 37.9%, *P* < .001). Multivariate regression analysis showed that, compared with the OMA phenotype, the DIE phenotype was an independent negative predictor for significant pain relief (odds ratio = 0.67, 95% confidence interval: 0.43–0.99, *P* = .046). DNG is significantly effective for all 3 endometriosis phenotypes, effectively relieving pain, reducing lesion size, and improving quality of life. However, patients with the DIE phenotype, due to the inherently more severe nature of their disease, have a relatively lower likelihood of achieving significant pain relief and a higher incidence of adverse reactions, necessitating special attention in clinical management.

## 1. Introduction

Endometriosis is a common, estrogen-dependent chronic inflammatory gynecological disorder, defined by the presence of endometrial-like tissue outside the uterine cavity. It affects approximately 10% of women of reproductive age globally.^[1]^ The condition is associated with debilitating symptoms, including dysmenorrhea, chronic pelvic pain, and infertility, which significantly impair patients’ quality of life and impose a substantial socioeconomic burden.^[2]^

A key characteristic of endometriosis is its profound heterogeneity in clinical presentation and pathological features. Based on anatomical localization, the disease is primarily classified into 3 distinct phenotypes: ovarian endometrioma (OMA), often referred to as “chocolate cysts”; superficial peritoneal endometriosis (SUP), characterized by superficial implants on the pelvic peritoneum; and deep infiltrating endometriosis (DIE), which involves lesions penetrating more than 5 mm beneath the peritoneal surface and can infiltrate vital pelvic structures.^[3]^ Growing evidence suggests that these phenotypes may represent distinct disease entities with potentially different underlying pathogenesis, molecular profiles, clinical behavior, and progression patterns, rather than mere morphological variations of a single disorder.^[4,5]^ Notably, DIE is frequently associated with more severe pain symptoms, higher revised American Society for Reproductive Medicine (rASRM) scores, and greater surgical complexity.^[6]^

Dienogest (DNG), a highly selective synthetic progestin, has emerged as a first-line option for the long-term medical management of endometriosis.^[7,8]^ It exerts its therapeutic effects through central and peripheral mechanisms, effectively controlling the progression of endometriotic lesions and alleviating pain. While the overall efficacy and safety of DNG in the general endometriosis population are well-established, a critical and less explored clinical question remains: whether this standardized pharmacotherapy yields comparable therapeutic responses across different phenotypic manifestations of the disease.

Currently, research specifically investigating phenotype-specific responses to DNG is limited, and existing findings are sometimes inconsistent. Some studies hypothesize that the predominantly fibromuscular composition of DIE nodules may confer a different drug responsiveness compared with the cystic and inflammation-driven nature of OMA lesions.^[9]^ Elucidating these potential differential responses is crucial for advancing personalized treatment strategies, enabling clinicians to set realistic therapeutic expectations, and optimizing patient management and resource allocation.

Therefore, this study aimed to address the following scientific questions: whether the therapeutic response to DNG differs among patients with OMA, SUP, and DIE phenotypes; which phenotypic or clinical factors independently influence significant pain relief; and whether safety and quality-of-life outcomes vary across phenotypes. The innovation of this study lies in its large-sample, phenotype-specific comparative analysis in a real-world setting, providing clinically relevant evidence to support precision treatment strategies for endometriosis.

## 2. Materials and methods

### 2.1. Study design

#### 2.1.1. Study participants

This study was approved by the Ethics Committee of Tianjin Central Hospital of Gynecology Obstetrics. This study retrospectively collected a total of 501 patients diagnosed and treated for endometriosis at our hospital between January 2023 and January 2024. Based on phenotypic differences, they were divided into OMA, SUP, and DIE. According to the above classification criteria, the final included cases were 276 OMA cases (approximately 55.1%), 125 SUP cases (approximately 25.0%), and 100 DIE cases (approximately 19.9%). For patients with multiple types of lesions simultaneously, grouping was based on the principle of the most severe phenotype (DIE > OMA > SUP) to ensure mutually exclusive groups for statistical comparison.

#### 2.1.2. Phenotypic classification

Based on the lesion location and imaging/intraoperative findings, patients were divided into 3 phenotypes:

OMA: lesions mainly involving the ovaries, with imaging showing typical “chocolate cysts.”
SUP: lesions confined to the superficial peritoneal layer, commonly found on the pelvic peritoneum or the surface of the uterosacral ligaments.
DIE: lesions invading >5 mm beneath the peritoneum, potentially involving deep structures such as the rectouterine pouch, posterior vaginal fornix, bladder, or intestinal wall.

### 2.2. Inclusion and exclusion criteria

#### 2.2.1. Inclusion criteria

Definitive diagnosis: endometriosis confirmed by imaging (ultrasound or magnetic resonance imaging) and/or intraoperative pathology; classifiable lesions: based on imaging or surgical findings, could be clearly classified as ovarian (OMA), peritoneal (SUP), or deep infiltrating (DIE); consistent treatment: all received DNG (2 mg/day orally) monotherapy, with continuous medication for ≥6 months; complete data: had complete clinical data, imaging data, and follow-up records; age range: reproductive-aged women between 18 and 50 years; and no pregnancy plans: not pregnant during the treatment period and did not receive other hormonal therapy or assisted reproductive intervention.

#### 2.2.2. Exclusion criteria

Following are the exclusion criteria: those with incomplete imaging or pathological data, making clear classification impossible; those complicated with uterine malignancies, ovarian cancer, or other malignant reproductive system diseases; those concurrently using other hormonal drugs (such as gonadotropin-releasing hormone agonist [GnRH-a], oral contraceptives, and compound progestin preparations) or receiving surgical treatment; those with previous allergies to DNG or other progestin drugs; those complicated with severe systemic diseases (such as hepatic or renal insufficiency, thrombotic diseases, autoimmune diseases) affecting efficacy evaluation; those pregnant, lactating, or who became pregnant during the treatment period; and those with insufficient follow-up time or missing clinical data, making efficacy evaluation impossible.

### 2.3. Treatment methods

All patients received monotherapy with DNG, which may be commercially known as Visanne® or similar preparations. DNG is a highly selective progestin derivative that inhibits ovulation, reduces estrogen levels, and suppresses inflammatory responses and angiogenesis, effectively alleviating pain and inhibiting lesion growth.

#### 2.3.1. Administration regimen

Patients began oral administration of 2 mg DNG once daily after diagnosis, continuing without interruption for at least 6 months. For patients experiencing menstrual changes (such as breakthrough bleeding) during treatment, they were instructed to continue medication and were followed up for observation. No other hormonal medications (such as GnRH-a, contraceptives, medroxyprogesterone acetate, etc) or assisted reproductive therapies were used concurrently during the treatment period.

#### 2.3.2. Follow-up and compliance management

All patients were followed up before treatment, at 3 months of treatment, and at 6 months of treatment, recording symptom changes, physical signs, laboratory indicators, and imaging findings.

Pain assessment: Visual analog scale (VAS) was used to assess dysmenorrhea, pelvic pain, dyspareunia, etc.

Imaging follow-up: Ultrasound or magnetic resonance imaging was used to measure lesion size, cyst volume, or nodule diameter.

Laboratory testing: Changes in serum CA125 levels were detected.

Adverse reaction monitoring: Headaches, breast tenderness, weight changes, irregular vaginal bleeding, mood swings, and other conditions occurring during treatment were recorded.

If patients experienced severe adverse reactions (such as persistent heavy bleeding, significant abnormal liver function, etc), they could discontinue medication under physician guidance and be excluded from subsequent analysis. Patient compliance was confirmed through medication collection records and follow-up inquiries; if medication was interrupted for more than 14 days, it was defined as poor compliance and excluded from analysis.

#### 2.3.3. Efficacy assessment time points

Efficacy was primarily compared based on clinical and imaging data before treatment and after 6 months of treatment. For some patients who completed 12 months of follow-up, long-term efficacy and recurrence were recorded for additional analysis.

### 2.4. Statistical analysis

All statistical analyses were performed using professional statistical software (such as SPSS 26.0 [IBM Corporation, Armonk] or R 4.2.0 [The R Foundation for Statistical Computing, Vienna, Austria]), with the significance level set at α = 0.05. Normally distributed continuous data are described as mean ± standard deviation (*x̄* ± *s*), such as age, body mass index (BMI), and VAS scores; non-normally distributed continuous data are described as median and interquartile range (M [IQR]), such as disease duration and CA125 levels; categorical data are presented as numbers and percentages (n [%]). For comparisons of baseline characteristics among the 3 groups, normally distributed continuous variables were analyzed using one-way analysis of variance (ANOVA), non-normally distributed variables using the Kruskal–Wallis *H* test, and categorical variables using the chi-square test or Fisher exact test. To evaluate efficacy, continuous variables at different time points before and after treatment (such as VAS scores, 36-Item Short Form Health Survey [SF-36] scores) were compared using repeated measures ANOVA or generalized estimating equations; changes before and after treatment (such as ΔVAS, lesion reduction rate) were compared between groups using one-way ANOVA or the Kruskal–Wallis *H* test; differences in categorical efficacy indicators (such as pain improvement rate, incidence of adverse reactions) between groups were analyzed using the chi-square test. Additionally, to explore independent factors influencing significant pain relief (defined as a ≥50% reduction in VAS score), we included clinical variables, such as phenotype, baseline VAS score, and total rASRM score, in a multivariate logistic regression analysis, with results expressed as adjusted odds ratios (OR) and their 95% confidence intervals.

## 3. Results

### 3.1. Baseline characteristics of endometriosis patients with different phenotypes

A total of 501 patients were enrolled, comprising 276 (55.1%) with OMA, 125 (25.0%) with SUP, and 100 (19.9%) with DIE (Table 1). No statistically significant differences were observed among the 3 groups regarding age, BMI, or marital and obstetric history (*P* > .05). However, significant differences were found in disease duration, rASRM stage and score, CA125 levels, and pain intensity (*P* < .001). Patients in the DIE group had the longest disease duration, the highest rASRM scores and CA125 levels, the most severe dysmenorrhea, and higher rates of concurrent adenomyosis, previous surgical history, and anemia, indicating a more severe disease status in this group.

**Table 1:** Baseline characteristics of patients with different phenotypes of endometriosis.

Variable	OMA group (n = 276)	SUP group (n = 125)	DIE group (n = 100)	Statistic (*F*/χ^2^)	*P* value
Age (years, *x̄* ± *s*)	33.2 ± 5.8	32.6 ± 6.1	34.8 ± 5.9	3.42	.034*
BMI (kg/m^2^, *x̄* ± *s*)	22.4 ± 2.9	22.1 ± 3.1	22.7 ± 2.8	0.88	.415
Disease duration (years, median [IQR])	3.0 (2.0–5.0)	2.5 (1.5–4.0)	5.0 (3.0–7.0)	18.72	<.001*
Marital status (married, n/%)	205 (74.3%)	86 (68.8%)	82 (82.0%)	5.64	.06
Parity (parous, n/%)	156 (56.5%)	68 (54.4%)	55 (55.0%)	0.17	.92
rASRM stage (I–II/III–IV, n)	78/198	70/55	12/88	89.74	<.001*
rASRM total score (points, *x̄* ± *s*)	46.8 ± 21.5	28.2 ± 15.4	62.1 ± 23.7	54.72	<.001*
Baseline CA125 (U/mL, median [IQR])	72 (45–110)	56 (35–82)	118 (80–168)	52.3	<.001*
VAS for dysmenorrhea (score, *x̄* ± *s*)	6.3 ± 1.7	5.4 ± 1.5	7.2 ± 1.8	34.21	<.001*
Concomitant adenomyosis (n/%)	78 (28.3%)	22 (17.6%)	42 (42.0%)	18.47	<.001*
History of pelvic surgery (n/%)	102 (37.0%)	33 (26.4%)	54 (54.0%)	22.86	<.001*
Hemoglobin (g/L, *x̄* ± *s*)	123.6 ± 12.4	125.1 ± 11.6	118.4 ± 13.2	12.1	<.001*
Anemia (Hb < 120 g/L, n/%)	102 (37.0%)	38 (30.4%)	52 (52.0%)	16.27	<.001*

BMI = body mass index, DIE = deep infiltrating endometriosis, Hb = hemoglobin, IQR = interquartile range, OMA = ovarian endometrioma, rASRM = revised American Society for Reproductive Medicine, SUP = superficial peritoneal endometriosis, VAS = visual analog scale.

* Indicates *P* < .05.

### 3.2. Changes in pain scores before and after DNG treatment in endometriosis patients with different phenotypes

Significant differences in dysmenorrhea and chronic pelvic pain (VAS) scores were observed among the 3 groups before treatment (*P* < .001), with the DIE group exhibiting the most severe pain, followed by the OMA group and the SUP group showing the mildest pain (Table 2). After 3 and 6 months of DNG treatment, pain scores significantly decreased from baseline in all 3 groups (*P* < .001). At the 6-month mark, the reduction in dysmenorrhea VAS scores (ΔVAS) showed statistically significant differences among groups (*P* = .008), with the SUP group demonstrating a significantly smaller improvement (−3.2 ± 1.1) compared with the OMA (−3.7 ± 1.2) and DIE (−3.7 ± 1.3) groups. Similarly, the reduction in chronic pelvic pain VAS scores also showed significant intergroup differences (*P* = .006), with the SUP group showing the least improvement (−2.1 ± 1.0), while the DIE group exhibited the greatest improvement (−2.9 ± 1.2). However, no statistically significant differences were found among the 3 groups in the rates of significant pain improvement, defined as a ≥50% reduction in VAS scores (dysmenorrhea: 71.0%–78.3%, *P* = .281; chronic pelvic pain: 63.2%–68.1%, *P* = .650).

**Table 2:** Changes in VAS scores for pain before and after dienogest treatment among different endometriosis phenotypes.

Pain type and time point	OMA group (n = 276)	SUP group (n = 125)	DIE group (n = 100)	Statistic (*F*)	*P* value
Dysmenorrhea VAS (score, *x̄* ± *s*)					
Before treatment	6.3 ± 1.7	5.4 ± 1.5	7.2 ± 1.8	34.21	<.001*
3 mo	3.9 ± 1.4	3.1 ± 1.2	4.7 ± 1.5	29.34	<.001*
6 mo	2.6 ± 1.1	2.2 ± 0.9	3.5 ± 1.3	26.1	<.001*
△VAS (6 mo–baseline)	−3.7 ± 1.2	−3.2 ± 1.1	−3.7 ± 1.3	4.85	.008*
Chronic pelvic pain VAS (score, *x̄* ± *s*)					
Before treatment	4.8 ± 1.6	3.9 ± 1.4	5.8 ± 1.8	28.75	<.001*
3 mo	3.1 ± 1.2	2.6 ± 1.0	3.9 ± 1.3	22.1	<.001*
6 mo	2.1 ± 0.9	1.8 ± 0.8	2.9 ± 1.0	24.65	<.001*
△VAS (6 mo–baseline)	−2.7 ± 1.1	−2.1 ± 1.0	−2.9 ± 1.2	5.12	.006*
Significant pain improvement (VAS reduction ≥50%, n/%)					
Dysmenorrhea	216 (78.3%)	94 (75.2%)	71 (71.0%)	2.54	.281
Chronic pelvic pain	188 (68.1%)	79 (63.2%)	64 (64.0%)	0.86	.65

DIE = deep infiltrating endometriosis, OMA = ovarian endometrioma, SUP = superficial peritoneal endometriosis, VAS = visual analog scale.

* Indicates *P* < .05.

### 3.3. Imaging changes in lesions before and after DNG treatment

After 6 months of DNG treatment, lesions in all 3 groups showed a significant reduction compared with baseline (Table 3). In the OMA group, the mean diameter of ovarian cysts decreased from 5.8 ± 2.1 cm to 3.0 ± 1.5 cm. In the SUP group, the area of peritoneal lesions reduced from 4.2 ± 1.6 cm^2^ to 2.7 ± 1.2 cm^2^. In the DIE group, the nodule diameter decreased from 17.4 ± 6.5 mm to 10.8 ± 5.2 mm. The overall lesion reduction rates were (47.5 ± 18.2)% in the OMA group, (39.4 ± 16.7)% in the SUP group, and (37.9 ± 19.5)% in the DIE group, with statistically significant differences among the groups (*P* < .001).

**Table 3:** Imaging changes before and after dienogest treatment.

Indicator	OMA group (n = 276)	SUP group (n = 125)	DIE group (n = 100)	Statistic (*F*/χ^2^)	*P* value
Baseline lesion size	5.8 ± 2.1 cm (OMA cyst)	4.2 ± 1.6 cm^2^ (peritoneal area)	17.4 ± 6.5 mm (nodule)	–	–
After 6 mo	3.0 ± 1.5 cm	2.7 ± 1.2 cm^2^	10.8 ± 5.2 mm	–	–
Shrinkage rate (%)	47.5 ± 18.2	39.4 ± 16.7	37.9 ± 19.5	12.84	<.001*

DIE = deep infiltrating endometriosis, OMA = ovarian endometrioma, SUP = superficial peritoneal endometriosis.

* Indicates *P* < .05.

### 3.4. Changes in serum CA125 levels before and after DNG treatment

Before treatment, significant differences in CA125 levels were observed among the 3 groups, with the DIE group showing the highest levels (118 [80–168] U/mL), followed by the OMA group (72 [45–110] U/mL) and the SUP group the lowest (56 [35–82] U/mL) (*P* < .001), as presented in Table 4. After 6 months of DNG treatment, CA125 levels significantly decreased in all groups (*P* < .001). The posttreatment median values were 34 (22–56) U/mL in the OMA group, 28 (18–42) U/mL in the SUP group, and 60 (38–95) U/mL in the DIE group. The magnitude of CA125 reduction was similar across the 3 groups, with an average decrease of approximately 50% (*P* = .345 for intergroup difference).

**Table 4:** Changes in serum CA125 levels before and after dienogest treatment.

Indicator	OMA group (n = 276)	SUP group (n = 125)	DIE group (n = 100)	Statistic (*F*/χ^2^)	*P* value
Baseline CA125 (U/mL, median [IQR])	72 (45–110)	56 (35–82)	118 (80–168)	52.3	<.001*
After 6 mo	34 (22–56)	28 (18–42)	60 (38–95)	40.26	<.001*
Reduction rate (%)	−52.8 ± 21.3	−49.7 ± 20.8	−48.9 ± 23.5	1.07	.345

DIE = deep infiltrating endometriosis, IQR = interquartile range, OMA = ovarian endometrioma, SUP = superficial peritoneal endometriosis.

* Indicates *P* < .05.

### 3.5. Comparison of adverse reactions and compliance during DNG treatment

During the treatment course, adverse reactions were reported in a certain proportion of patients across the different phenotypes (Table 5). The overall incidence of adverse events (AEs) was 46.4% in the OMA group, 38.4% in the SUP group, and 58.0% in the DIE group, with a statistically significant difference (*P* = .004). Common adverse reactions included breakthrough bleeding (23.2%, 16.0%, 32.0%), breast tenderness (14.9%, 11.2%, 20.0%), weight gain ≥2 kg (13.0%, 8.0%, 17.0%), and mood swings (12.0%, 8.8%, 18.0%). The incidences of breakthrough bleeding, weight gain, and mood swings were higher in the DIE group (all *P* < .05). The proportion of treatment discontinuation due to adverse reactions was low (4.3%–9.0%), with no statistically significant difference among the groups (*P* = .053). Overall compliance was good, with medication adherence rates exceeding 90% in all 3 groups.

**Table 5:** Adverse effects and treatment adherence during dienogest therapy.

Adverse effect/adherence	OMA (n = 276)	SUP (n = 125)	DIE (n = 100)	χ^2^/*F*	*P* value
Breakthrough bleeding	64 (23.2%)	20 (16.0%)	32 (32.0%)	10.58	.005*
Breast tenderness	41 (14.9%)	14 (11.2%)	20 (20.0%)	4.89	.087
Weight gain ≥ 2 kg	36 (13.0%)	10 (8.0%)	17 (17.0%)	6.72	.035*
Mood fluctuation	33 (12.0%)	11 (8.8%)	18 (18.0%)	6.48	.039*
Any adverse event (≥1)	128 (46.4%)	48 (38.4%)	58 (58.0%)	10.95	.004*
Discontinuation due to AE	12 (4.3%)	4 (3.2%)	9 (9.0%)	5.87	.053
Good adherence (%)	93.5	95.2	90	–	–

AE = adverse event, DIE = deep infiltrating endometriosis, OMA = ovarian endometrioma, SUP = superficial peritoneal endometriosis.

* Indicates *P* < .05.

### 3.6. Changes in SF-36 quality-of-life scores before and after DNG treatment

Prior to treatment, differences in SF-36 total scores were observed among patients with different phenotypes (*P* < .001), with the DIE group exhibiting the lowest scores, while the OMA and SUP groups showed relatively higher scores (Table 6). After 6 months of DNG treatment, quality-of-life scores improved significantly in all 3 groups (*P* < .001). However, the magnitude of improvement (Δ) in quality-of-life scores demonstrated significant differences among the groups (*P* = .018). Patients in the DIE group showed the most pronounced improvement in SF-36 scores (+17.5 ± 8.5), which was significantly greater than the improvements observed in the OMA group (+14.3 ± 8.2) and the SUP group (+13.7 ± 7.9). These results suggest that although the absolute posttreatment quality-of-life scores remained the lowest in the DIE group, these patients derived the greatest relative benefit from the treatment.

**Table 6:** SF-36 Health Survey scores before and after 6 months of dienogest therapy.

Time point	OMA group (n = 276)	SUP group (n = 125)	DIE group (n = 100)	*F* value	*P* value
Baseline	60.6 ± 12.7	62.9 ± 12.1	53.9 ± 13.5	8.24	<.001*
After 6 mo	74.9 ± 11.8	76.6 ± 11.3	71.4 ± 12.4	5.36	.005*
Change (Δ, mean ± SD)	+14.3 ± 8.2	+13.7 ± 7.9	+17.5 ± 8.5	4.15	.018*

DIE = deep infiltrating endometriosis, OMA = ovarian endometrioma, SD = standard deviation, SF-36 = 36-Item Short Form Health Survey, SUP = superficial peritoneal endometriosis.

* Indicates *P* < .05.

### 3.7. Multivariate logistic regression analysis of significant pain relief

Using significant pain improvement (defined as a ≥50% reduction in VAS score) as the dependent variable, a multivariate logistic regression analysis was conducted, incorporating clinically relevant factors (Table 7). The results identified phenotype, baseline VAS score, total rASRM score, CA125 level, presence of adenomyosis, and hemoglobin level as significant influencing factors. Compared with the OMA group, patients in the DIE group had a lower likelihood of achieving significant pain relief (OR = 0.67, 95% confidence interval: 0.43–0.99, *P* = .046), while no statistically significant difference was observed for the SUP group. A higher baseline VAS score was associated with a greater probability of significant improvement (OR = 1.22, *P* < .001). Conversely, both higher rASRM scores and elevated CA125 levels were associated with reduced treatment efficacy (*P* < .05). Furthermore, patients with coexisting adenomyosis had a lower probability of significant improvement (OR = 0.72, *P* = .044), while higher hemoglobin levels were associated with better treatment outcomes (OR = 1.10, *P* = .049). Age, BMI, disease duration, and previous surgical history did not significantly affect treatment efficacy (*P* > .05).

**Table 7:** Multivariable logistic regression for significant pain response (VAS reduction ≥50%).

Variable	Adjusted OR (95% CI)	*P* value
Phenotype		
OMA (reference)	–	–
SUP vs OMA	0.94 (0.62–1.43)	.78
DIE vs OMA	0.67 (0.43–0.99)	.046*
Baseline VAS (per 1-point increase)	1.22 (1.11–1.34)	<.001*
rASRM total score (per 10-point increase)	0.90 (0.83–0.98)	.014*
CA125 (per 50 U/mL increase)	0.94 (0.89–0.99)	.023*
Adenomyosis (yes vs no)	0.72 (0.52–0.99)	.044*
Prior pelvic surgery (yes vs no)	0.88 (0.65–1.20)	.41
Age (per 5-yr increase)	0.96 (0.85–1.08)	.49
BMI (per 1 kg/m^2^ increase)	0.98 (0.93–1.03)	.44
Hemoglobin (per 10 g/L increase)	1.10 (1.00–1.21)	.049*
Disease duration (per 1-yr increase)	0.97 (0.93–1.02)	.23

BMI = body mass index, CI = confidence interval, DIE = deep infiltrating endometriosis, OMA = ovarian endometrioma, OR = odds ratio, rASRM = revised American Society for Reproductive Medicine, SUP = superficial peritoneal endometriosis, VAS = visual analog scale.

* Indicates *P* < .05.

## 4. Discussion

Previous studies have consistently demonstrated the efficacy of DNG in alleviating pain symptoms and reducing lesion size in patients with endometriosis. Systematic reviews and pooled analyses have confirmed its favorable safety profile and suitability for long-term management. However, most existing studies have evaluated endometriosis as a homogeneous disease entity, without stratifying patients according to anatomical or phenotypic classifications.

Emerging evidence suggests that OMA, SUP, and DIE may represent biologically and clinically distinct subtypes, characterized by different histological features, inflammatory profiles, and pain mechanisms. Nevertheless, data comparing the therapeutic response with DNG across these phenotypes remain limited and inconsistent. Our study addresses this gap by systematically comparing pain relief, lesion regression, quality-of-life improvement, and AE profiles among these 3 phenotypes in a large, real-world cohort, thereby providing phenotype-oriented evidence to support individualized clinical decision-making.

Regarding pain relief, our results reveal multiple dimensions of DNG efficacy. On the one hand, VAS scores decreased significantly after treatment in all 3 groups, and the proportion of patients achieving significant improvement (≥50% reduction in VAS) did not differ among groups, strongly demonstrating the universal effectiveness of DNG as a first-line drug.^[7,8]^ On the other hand, in-depth analysis of the magnitude of pain improvement (ΔVAS) showed that the improvement in the SUP phenotype was significantly smaller than that in the OMA and DIE phenotypes. This finding may stem from the milder baseline pain in SUP patients, resulting in an inherently limited absolute scope for improvement. More insightfully, multivariate logistic regression analysis revealed that, after adjusting for confounding factors such as baseline pain and disease severity, the DIE phenotype itself was an independent negative predictor of significant pain relief (OR = 0.67). This finding holds significant pathophysiological importance. It suggests that the relative drug resistance of DIE lesions to DNG may not only originate from their more severe clinical manifestations but also be related to their unique histological characteristics. For instance, a higher proportion of fibrosis and smooth muscle hyperplasia within the lesions might limit drug penetration and efficacy.^[10,11]^

In the assessment of imaging findings and quality of life, we observed intriguing patterns of phenotypic variation. The OMA group demonstrated the highest lesion reduction rate, which aligns with its cystic structural characteristics, as the medication effectively reduces intracystic bleeding, leading to a rapid decrease in cyst volume.^[12]^ By contrast, SUP and DIE lesions contain substantial established fibrotic architecture, limiting the drug’s ability to resolve their structure.^[13,14]^ However, the pattern of quality-of-life improvement was reversed: although the absolute SF-36 scores in the DIE group remained the lowest posttreatment, the magnitude of improvement (Δ) was significantly greater than that in the other 2 groups. This yields an important clinical implication: for DIE patients with the highest disease burden, even if imaging improvement is less than ideal, the subjective quality-of-life benefit they derive from treatment may be the most substantial. This discrepancy between patient-reported outcomes and objective indicators underscores the importance of a comprehensive efficacy evaluation.

Regarding safety, the total incidence of AEs was significantly higher in the DIE group. The reasons for this are not entirely clear, but we speculate it might be related to the higher baseline disease burden, more extensive lesion distribution, or an inherently heightened sensitivity to hormonal fluctuations in DIE patients.^[8,15]^ Nevertheless, the discontinuation rates did not differ significantly among the 3 groups, suggesting that with adequate doctor-patient communication and expectation management, long-term use of DNG remains feasible in DIE patients.^[16,17]^

The findings of this study provide direct evidence for the individualized clinical management of endometriosis. For patients with the SUP phenotype, it should be communicated that the potential for a large absolute reduction in pain scores might be limited, yet the proportion achieving clinically meaningful improvement is comparable with other phenotypes. For patients with the DIE phenotype, multidimensional treatment expectations should be set: they should be informed about the relatively lower likelihood of achieving complete pain relief and the higher risk of adverse reactions, while also emphasizing the potentially greatest degree of benefit in terms of quality of life. For the latter group, considering combination therapy with more potent agents such as GnRH-a during the initial treatment phase to achieve rapid symptom control could be an option.^[18,19]^

This study has several limitations. Its retrospective design and single-center nature inevitably introduce selection bias. The pain scores used in efficacy assessment are subjective, and the 6-month follow-up period is insufficient to reveal long-term outcomes. Future research should aim to validate these findings through prospective designs, extended follow-up durations, and incorporation of histological and molecular markers. Several potential confounding factors should be considered when interpreting our findings. Baseline disease severity, differences in pain perception, coexistence of adenomyosis, and variations in lesion distribution may have influenced treatment response. Although multivariate regression analysis was performed to adjust for major confounders, residual confounding cannot be entirely excluded due to the retrospective nature of the study. Future research should adopt prospective cohort designs or multicenter studies with standardized assessment protocols to further validate and generalize these results.

In conclusion, this study confirms that DNG is significantly effective for all types of endometriosis, but different phenotypes exhibit distinct treatment response patterns. DIE faces greater challenges in achieving complete pain relief but shows significant benefit in quality-of-life improvement, SUP shows a relatively smaller magnitude of pain improvement, and OMA demonstrates the most pronounced effect in lesion reduction. These findings highlight the importance of considering disease phenotype in the treatment of endometriosis and provide new evidence for advancing precision medicine in this field.

## Author contributions

**Conceptualization:** Jixiao Liu, Dandan Wu, Jianli Sun.

**Funding acquisition:** Jixiao Liu.

**Data curation:** Jixiao Liu, Dandan Wu, Jianli Sun.

**Formal analysis:** Jixiao Liu, Dandan Wu, Jianli Sun.

**Investigation:** Jixiao Liu.

**Writing – original draft:** Jixiao Liu, Dandan Wu.

**Writing – review & editing:** Jixiao Liu, Dandan Wu.
